# Supplementary material for: Iraqi Parents’ Knowledge, Attitudes, and Practices towards Vaccinating Their Children: A Cross-Sectional Study
Source: Vaccines (Basel). 2022 May 22;10(5):820. doi: 10.3390/vaccines10050820 (PMC9144119; doi:10.3390/vaccines10050820)
Supplement: Supplementary file 1 [file vaccines-10-00820-s001.zip › vaccines-1743725-supplementary.pdf]

Table S1. Pilot study results

		Frequency (%) or Mean (± SD)
Sex	Male	17 (56.7%)
	Female	13 (43.3%)
Age	18-29 years	7 (23.3%)
	30-39 years	14 (46.7%)
	40 years or above	9 (30.0%)
Education	High school or less	5 (16.7%)
	Diploma	2 (6.7%)
	University student	6 (20.0%)
	Bachelor	11 (36.7%)
	Postgraduate	6 (20.0%)
	Less than 500$	12 (40.0%)
	500-1000$	10 (33.3%)
	More than 1000$	0 (0.0%)
Children	1	7 (23.3%)
	2	8 (26.7%)
	3	5 (16.7%)
	4 or more	10 (33.3%)
Children's sex	Girls	3 (10.0%)
	Boys	10 (33.3%)
	Boys and girls	17 (56.7%)
adherence to protective practice score		18.73 (± 4.21)
Knowledge score		13.03 (± 3.06)
Have you taken or planning to take COVID-19 vaccine?	Yes	27 (90.0%)
	No	3 (10.0%)
Willing to vaccinate children against COVID-19	No	17 (56.7%)
	Maybe	4 (13.3%)
	Yes	9 (30.0%)

Table S2. Knowledge towards COVID-19 and its vaccines (correct answers).

Knowledge determinants	Frequency (%)
**What are the symptoms of COVID19?**	
Fever	475 (96.7)
Chills	276 (56.2)
Diarrhea	365 (74.3)
Cough	432 (88.0)
Otitis media	290 (59.1)
Loss of smell and taste senses	442 (90.0)
No symptoms	366 (74.5)
**How COVID19 can be transmitted?**	
Drinking unclean water	300 (61.1)
Eating unclean food	274 (55.8)
Inhalation of respiratory droplets of infected person	458 (93.3)
Eating or touching wild animals	296 (60.3)
**What procedures do you think may prevent COVID-19 infection?**	
Wearing face masks	438 (89.2)
Washing hands with regular soap	407 (82.9)
Using detergents	435 (88.6)
Social distancing	450 (91.6)
Avoid touching face/mouth/nose/eyes	415 (84.5)
Avoid eating meat	357 (72.7)
Consume herbs	264 (53.8)
Rote of vaccine administration	317 (64.6)
Availability of medicine	294 (59.9)

Table S3. Adherence to protective practices against COVID-19.

What procedures have you taken to protect yourself from COVID19?	Mean (SD)	Never	Rarely	Sometimes	Most of the time	All the time
Wearing face masks	3.62 (1.04)	12 (2.4)	65 (13.2)	131 (26.7)	173 (35.2)	110 (22.4)
Washing hands with regular soap	4.22 (1.00)	18 (3.7)	16 (3.3)	48 (9.8)	166 (33.8)	243 (49.5)
Using detergents	3.73 (1.09)	19 (3.9)	45 (9.2)	126 (25.7)	159 (32.4)	142 (28.9)
Social distancing	3.76 (1.03)	16 (3.3)	33 (6.7)	139 (28.3)	166 (33.8)	137 (27.9)
Avoid touching face/mouth/nose/eyes	3.75 (1.11)	20 (4.1)	47 (9.6)	119 (24.2)	156 (31.8)	149 (30.3)

Note: SD: standard deviation.
